# Mitochondrial Involvement in Migration, Invasion and Metastasis

**DOI:** 10.3389/fcell.2019.00355

**Published:** 2019-12-20

**Authors:** Tatiana V. Denisenko, Anna S. Gorbunova, Boris Zhivotovsky

**Affiliations:** ^1^Faculty of Medicine, Lomonosov Moscow State University, Moscow, Russia; ^2^Institute of Environmental Medicine, Division of Toxicology, Karolinska Institute, Stockholm, Sweden

**Keywords:** cell death, invasion, metastasis, migration, mitochondria

## Abstract

Mitochondria in addition to be a main cellular power station, are involved in the regulation of many physiological processes, such as generation of reactive oxygen species, metabolite production and the maintenance of the intracellular Ca^2+^ homeostasis. Almost 100 years ago Otto Warburg presented evidence for the role of mitochondria in the development of cancer. During the past 20 years mitochondrial involvement in programmed cell death regulation has been clarified. Moreover, it has been shown that mitochondria may act as a switchboard between various cell death modalities. Recently, accumulated data have pointed to the role of mitochondria in the metastatic dissemination of cancer cells. Here we summarize the modern knowledge concerning the contribution of mitochondria to the invasion and dissemination of tumor cells and the possible mechanisms behind that and attempts to target metastatic cancers involving mitochondria.

## Introduction

**FIGURE 1:**
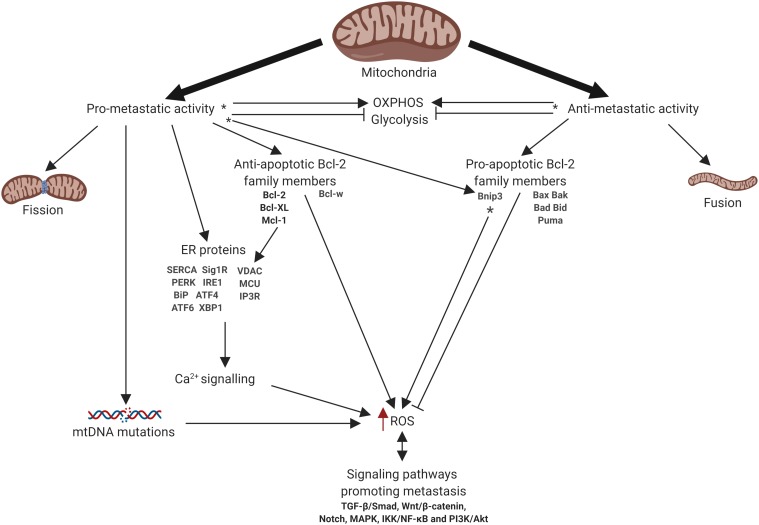
Schematic representation of mitochondrial involvement in metastasis. Arrows or blunt ends indicate activation or inhibition, respectively. Red arrow indicates increased level. ^∗^ - function depends on the tumor type. OXPHOS, oxidative phosphorylation; ER, endoplasmic reticulum; ROS, reactive oxygen species. For details, see text. Figure is created using BioRender.

Mitochondria are intracellular organelles that produce the majority of the energy in the cells, providing synthesis of ATP by oxidative phosphorylation (OXPHOS) (Mitchell, 1961). Beyond energy production mitochondria have multiple functions including the generation of reactive oxygen species (ROS), metabolite production, the regulation of intracellular Ca^2+^ homeostasis and modulation of cell death pathways. Additionally, mitochondria contribute to the regulation of signaling pathways linked to the cell proliferation, differentiation, and many others (Porporato et al., 2018). The multiple functions of mitochondria allow cells to adapt to the changing of environment, including the availability of nutrients and oxygen, making them perfect stress sensors (Vyas et al., 2016). These functions also determine the crucial role of mitochondria in development and progression of cancer. Indeed, mitochondria may drive tumor progression through adaptation to changing metabolic demand, contributing to chemoresistance, and regulating cell death pathways (Gogvadze et al., 2008). Furthermore, mitochondria have been shown to be linked to the metastatic dissemination of cancer cells. Importantly, mitochondrial turnover, i.e., fission/fusion, is deeply involved in the regulation of different mitochondrial functions and metastatic cascade. However, the role of mitochondrial dynamics in cancer cell invasion and metastasis remains highly controversial. Here we took an attempt to summarize the present knowledge about the functions of mitochondria that contribute to the metastatic dissemination and invasion including mitochondrial dynamics, cell death, oxidative stress, metabolism and bioenergetics, Ca^2+^ signaling, and mtDNA (Figure 1). Additionally, we highlight the existing therapy approaches to target metastatic cancers involving mitochondria.

## Mitochondria and Migration

Metastasis is one of the main cause of cancer patients’ death. Metastatic dissemination is characterized by cell detachment from the primary tumor mass, further migration through blood and lymphatic vessels and colonization of different tissues. The metastatic cascade can be subdivided into different stages, including local invasion, intravasation, survival in the circulation, extravasation, survival at a second site and finally outgrowth at this site. The epithelial to mesenchymal transition (EMT) is a biological phenomenon occurring during embryonic development but also associated with cancer metastasis (Chaffer and Weinberg, 2011). During EMT cancer cells lose their epithelial features and temporally acquire mesenchymal characteristics which allow them to migrate from the original site in order to colonize different tissues.

Epithelial to mesenchymal transition is regulated by various molecular pathways including TGF-β/Smad, Wnt/β-catenin, Notch, mitogen-activated protein kinase (MAPK), IKK/NF-κB and PI3K/Akt (Lamouille et al., 2014), cytokines (e.g., TGF-β and EGF) and transcription factors Snail (Snai1), Slug (Snai2), Twist (helix-loop-helix factor), and ZEB1/2 (zinc-finger E-box-binding homeobox) (Lu and Kang, 2019), etc. Overexpression of EMT transcriptional factors leads to the downregulation of epithelial markers and Tight Junction proteins, such as E-cadherin, occludin, and claudins, which in turn results in the loose of apical cell polarity (Lu and Kang, 2019). On the other hand, EMT activation provides the upregulation of mesenchymal markers: N-cadherin, vimentin, and fibronectin. In addition, EMT is accompanied by increased expression of matrix metalloproteinases (MMPs) and urokinase plasminogen activator (uPA), which contribute to the degradation of the extracellular matrix (ECM) and the basal membrane of epithelial tissue (Lu and Kang, 2019). These events lead to the loss of cell-cell and cell matrix adhesion contacts and an increase in cell motility and cell migration, which are the hallmarks of metastasis (Chaffer and Weinberg, 2011).

### Mitochondrial Dynamics and Migration

Mitochondria have been shown to contribute to carcinogenesis including metastatic dissemination and EMT by different mechanisms. Being extremely dynamic organelles mitochondria continuously change their morphology undergoing fission (fragmentation) and fusion (elongation). These processes are regulated by highly conserved guanosine triphosphatases (GTPases) (Senft and Ronai, 2016). Fission is controlled by cytosolic dynamin-related protein 1 (Drp1), which is recruited to mitochondria by adapter proteins, including mitochondrial fission factor (Mff) and mitochondrial dynamics proteins of 49 and 51 kDa (Mid49/51), where it forms oligomeric ring structures and executes mitochondrial fission. Fusion is mediated by two GTPases in the outer mitochondrial membrane (OMM), i.e., mitofusins 1 and 2 (Mfn1, 2), whereas the inner mitochondrial membrane (IMM) fusion is promoted by the cristae-shaping protein Opa1 (Losón et al., 2013). In cancer cells, mitochondrial fission/fusion is unbalanced due to the mitochondrial dysfunction (Srinivasan et al., 2017). Several studies have demonstrated that increased fission and/or reduced fusion are associated with malignant transformation in different types of cancer (Senft and Ronai, 2016). Furthermore, upregulation of mitochondrial fission and increased expression of Drp1 was shown to promote cancer metastasis (Zhao et al., 2013; Sun et al., 2018a). For example, overexpression of Drp1 was detected in breast cancer metastatic cells compared to the non-metastatic, whereas silencing of Drp1 or overexpression of Mfn1 resulted in mitochondrial elongation and significantly suppressed the metastatic properties of breast cancer cells (Zhao et al., 2013). Similarly, increased mitochondrial fission was observed in hepatocellular carcinoma (HCC) metastatic cells. Comparison of levels of Drp1 in tumor samples and in the normal tissues revealed its higher expression in the former, which is associated with the promotion of tumor cell survival and metastasis formation (Sun et al., 2018a). In addition, downregulation of Drp1 inhibits glioma cells invasive properties affecting cytoskeleton remodeling through the RhoA/ROCK1 pathway (Yin et al., 2016). Recent data have also suggested the existence of a link between mitochondrial fission and hypoxia-induced migration. The inhibition of Drp1 by Mdivi-1 leads to the decreased migration induced by hypoxia (Han et al., 2015). Taken together, these studies provide the evidence that mitochondrial fission is required for cancer cell migration and to support the metastatic potential of cancer cells. In migrating cancer cells, mitochondria localize at the leading edge along microtubules, where the energy demand is higher, providing necessary supply (Senft and Ronai, 2016). Unfortunately, the mechanism involving Drp1 in regulation of the process of cancer metastasis remains not fully understood. Different studies suggest that fission is required for efficient redistribution of mitochondria, and the upregulation/activation of Drp1 is associated with the migration of cancer cells (Zhao et al., 2013; Senft and Ronai, 2016). Another study has shown that inhibition of mitochondrial fusion may abolish invasion of syntaphilin-depleted prostate adenocarcinoma cells. Syntaphilin suppresses mitochondrial dynamics, cancer cell dissemination *in vivo*. Moreover, its downregulation correlates with poor outcome of cancer patients. Thereby, the silencing of both Mfn1, 2 and syntaphilin abolished mitochondrial trafficking and abrogated the migratory response (Caino et al., 2016). Thus, mitochondrial dynamics are linked to cancer cell migration, and the relative contribution of fission or fusion depends on tumor type and molecular context.

### ROS Contributes to Migration and Metastasis

Reactive oxygen species constantly generated during the metabolic process and play a crucial role in the regulation of various cellular functions (Vyas et al., 2016). Mitochondrial electron transport chain (ETC) is the main source of ROS (Vyas et al., 2016). Complexes I and III are often regarded as the major sites of mitochondrial ROS (mtROS) production, but more recent studies indicate that at least ten other mitochondrial enzymes also contribute to ROS generation, including Complex II (Quinlan et al., 2013). The role of ROS in cancer remains highly controversial. First, in cancer cells a higher level of ROS is detected compared to their normal counterparts (Cannito et al., 2010; Vyas et al., 2016). A moderate increase of ROS level was shown to support cancer cell proliferation and migration and to activate different signaling pathways associated with cell survival, contributing to tumor growth and malignant transformation (Kumari et al., 2018). Indeed, the level of ROS has been shown to activate the PI3K pathway. The primary known ROS target in the PI3K pathway is phosphatase and tensin homolog (PTEN). ROS promote the inactivation of the tumor suppressor PTEN by oxidizing active-site cysteine residues, causing the formation of a disulfide bond, which prevents PTEN from inactivating the PI3K pathway (Lee et al., 2002; Sullivan and Chandel, 2014). Since ROS can inactivate protein tyrosine phosphatases through oxidation of cysteine residues, ROS may have many yet-to-be discovered effects on diverse, mitogen-activated pathways that are normally inhibited by phosphatases (Sullivan and Chandel, 2014). ROS can stimulate the phosphorylation of MAPK and extracellular signal-regulated kinase (ERK), cyclin D1 expression and JUN N-terminal kinase (JNK) activation, all of which are linked to tumor cell survival and growth (Gorrini et al., 2013). ROS may activate different processes associated with metastatic dissemination and invasion. They may be involved in cytoskeleton remodeling. The cell cytoskeleton is dynamic structure composed of microtubules and filaments. Cytoskeletal rearrangements are important for driving cell migration and invasion through the formation of different types of cellular protrusions including filopodia, lamellipodia, and invadopodia (Ridley, 2011). Recent studies have shown that Rac-mediated actin remodeling is attributed to increased O_2_^–^ levels (Jiang et al., 2017). Specifically, Rho activation leads to the filopodia formation, while induction of Rac contributes to the formation of lamellipodia (Kozma et al., 1995; Narumiya et al., 2009; Galadari et al., 2017). Another mechanism by which ROS may promote tumor cell invasion is by stimulation of the proteolytic degradation of ECM components such as glycosaminoglycan (GAG), contributing to metastatic dissemination (Galadari et al., 2017).

Increased ROS levels can activate different pathways that induce morphological changes associated with the EMT (Jiang et al., 2017). For example, increased ROS generation stimulates the acquisition of invasive properties by pancreatic cancer cells through the activation of NF-κB signaling. In turn, treatment with antioxidants leads to the suppression of EMT and attenuates metastasis (Shimojo et al., 2013). NF-κB signaling is strongly associated with the EMT process by promoting the expression of the main EMT-related transcription factors Snail, Slug, Twist1, and ZEB1/2, which is also involved in the disruption of the cell–cell junctions (Min et al., 2008; Jiang et al., 2017). Furthermore, NF-κB activation may contribute to the transcription of vimentin and MMPs such as MMP-2, MMP-9, to maintain the mesenchymal phenotype and promote tumor cell migration (Jiang et al., 2017). Another pathway involved in EMT and regulated by ROS is the transcription factor hypoxia inducible factor 1-alpha (HIF-1α) (Jiang et al., 2017), which is induced under hypoxic conditions and can stimulate cancer cell EMT by activating EMT-inducing transcription factors such as Twist, Snail and ZEB1/2 (Joseph et al., 2015; Zhang et al., 2015). Thus, NF-κB is activated under hypoxic conditions, and thereby, in the presence of hypoxia, may co-regulate many of EMT-linked transcription factors (D’Ignazio et al., 2017). Importantly, ROS accumulation leads to the stabilization of HIF-1 due to inhibition of the HIF-degrading enzyme prolyl hydroxylase (Comito et al., 2011).

There is a complex interplay between the level of ROS and the TGF-β signaling pathway exists, which is the one of the most important pathways involved in EMT regulation. It was reported that ROS mediate TGF-β-induced EMT in cancer (Corcoran and Cotter, 2013; Liu and Desai, 2015). ROS may affect the activation of TGF-β downstream effector Smad, while treatment with the ROS scavenger N-acetyl cysteine (NAC) abolishes Smad phosphorylation (Krstiæ et al., 2015). Additionally, ROS may regulate TGF-β activation through different signaling pathways as described above, including MAPK and NF-κB (Corcoran and Cotter, 2013). Conversely, TGF-β can induce ROS production by many alterations in mitochondrial functioning and antioxidant protection. For example, TGF-β affects ROS levels by blocking of ETC Complex IV and upregulation of NADPH oxidase 4 (NOX4) (Yoon et al., 2005). Further, it was revealed that TGF-β increases ROS levels inhibiting ETC Complex III (Jain et al., 2013). TGF-β also downregulates the synthesis of the antioxidant glutathione (GSH) and several antioxidant enzymes contributing to cellular redox misbalance and EMT-related processes, such as fibrosis (Liu and Desai, 2015). Using the mitochondria-targeted antioxidant SkQ1 was also shown that oxidative stress is implicated in EMT induced by TGF-β. In cervical carcinoma SiHa cells depletion of ROS leads to increase of E-cadherin and downregulation of Snail, the main negative regulator of E-cadherin (Shagieva et al., 2017). Similarly, pretreatment with the ROS scavenger carotenoid astaxanthin (AST) leads to the suppression of EMT and the production of inflammatory cytokines by mesothelial cells (Hara et al., 2017). Thus, TGF-β, as inducer of EMT, is likely to affect this process through the ROS production.

Reactive oxygen species accumulation may influence migration and metastasis of cancer cells through different mechanisms affecting cytoskeleton remodeling, ECM degradation and the activation of signaling pathways. However, in conditions of strong oxidative stress, ROS suppress metastatic dissemination, due to induction of cell death or cellular senescence (Piskounova et al., 2015). Furthermore, elevated ROS levels may activate antioxidant pathways (Vyas et al., 2016). Indeed, oncogenic K- Ras-, B- Raf-, and c-Myc-mediated pathways may downregulate ROS production through regulation of nuclear factor (erythroid-derived 2)-like 2 (Nrf2), one of the main regulators of the antioxidant response (Vyas et al., 2016). Nrf2 provides a transcriptional activation of several genes involved in glutathione (GSH) synthesis. It promotes tumorigenesis contributing to the cancer cell protection against oxidative stress and chemotherapeutic agents (Rojo de la Vega et al., 2018). Recent data have demonstrated that Nrf2 activation can stimulate cancer cell migration and metastasis and Nrf2 deletion attenuates metastatic potential breast cancer cells suppressing RhoA GTPases activity (Zhang et al., 2016). Altogether, these observations demonstrate that the role of ROS in tumor progression and metastasis remains highly controversial. It has been suggested that tumors should maintain the ROS at a definite level in order to sustain their growth and metastasis without causing cytotoxicity (Vyas et al., 2016). Moreover, for tumor promotion it also necessary to provide the right balance between ROS production and antioxidants.

### Mitochondrial DNA Mutations Contribute to the Migration and Metastasis

It is known that mtDNA mutations can contribute to tumor initiation and progression (Vyas et al., 2016). Variations in copy number of mtDNA are associated with tumorigenesis and depend on tumor type (Sun et al., 2018b). Thus, decreased copy number of mtDNA was detected in breast cancer, HCC, non-small cell lung cancer (NSCLC) and gastric cancer (Mambo et al., 2005). On the other hand, increased copy number of mtDNA was found in prostate, head and neck, and colorectal cancers (Sun et al., 2018b). Mutations and variations in mtDNA content might be associated with regulation of the metastatic properties of tumor cells (Ishikawa et al., 2008). Replacement of mtDNA from a highly metastatic to a poorly metastatic cell line led to an increase in the metastatic potential in the recipient cell line (Ishikawa et al., 2008). mtDNA mutations are also associated with EMT of cancer cells. Indeed, EMT induced by TGF-β leads to an increase of mtDNA copy number in NSCLC cells (Xu and Lu, 2015). Conversely, knockdown of mitochondrial transcription factor A (TFAM) leads to a decrease in mtDNA copy number, upregulation of E-cadherin expression, and suppression of cell migration rate in esophageal squamous cell carcinoma (Lin et al., 2012). Increased mtDNA content in this type of tumor is associated with the higher energy required for EMT. Furthermore, mtDNA mutations contribute to the acquisition of an aggressive phenotype in oncocytic thyroid tumors leading to their bioenergetic crisis (De Luise et al., 2017). As a consequence, mitochondrial dysfunction may lead to the activation of glycolysis (Smolková et al., 2011). Thus, oxygen deprivation may provide positive selective pressure for cancer cells carrying damaging mtDNA mutations. However, another study provided the evidence that EMT could also be induced in mtDNA-depleted cells. Indeed, it has been demonstrated that TGF-β–induced EMT occurs in mitochondria-depleted cell lines leading to the stimulation of invasive properties through activation of Raf/MAPK (Naito et al., 2008). These data are consistent with the observation that in human mammary epithelial cells (hMECs) a decrease in mtDNA copy number promotes calcineurin-mediated mitochondrial retrograde signaling, which initiates EMT (Guha et al., 2014). Likewise, reduction of mtDNA content by suppression of mitochondrial pyrimidine nucleotide carrier 1 (PNC1), which is responsible for mitochondrial DNA replication, leads to EMT induction in hMECs (Favre et al., 2010). A recent study has revealed that increased mtDNA copy number may sustain tumor progression and metastasis by upregulating OXPHOS function in cancer cells that rely on mitochondrial OXPHOS. On the other hand, in cancer cells that depend on glycolytic type of metabolism reduction of mtDNA was shown to promote proliferation and chemoresistance (Sun et al., 2018b). Summing up, mtDNA mutations and variations of mtDNA copy number are associated with EMT, increased invasiveness and metastasis in different types of cancer. The opposite role of mtDNA content in cancer progression and metastatic dissemination depends on metabolic pattern of different types of cancer.

## Bcl-2 and Metastasis

**FIGURE 2:**
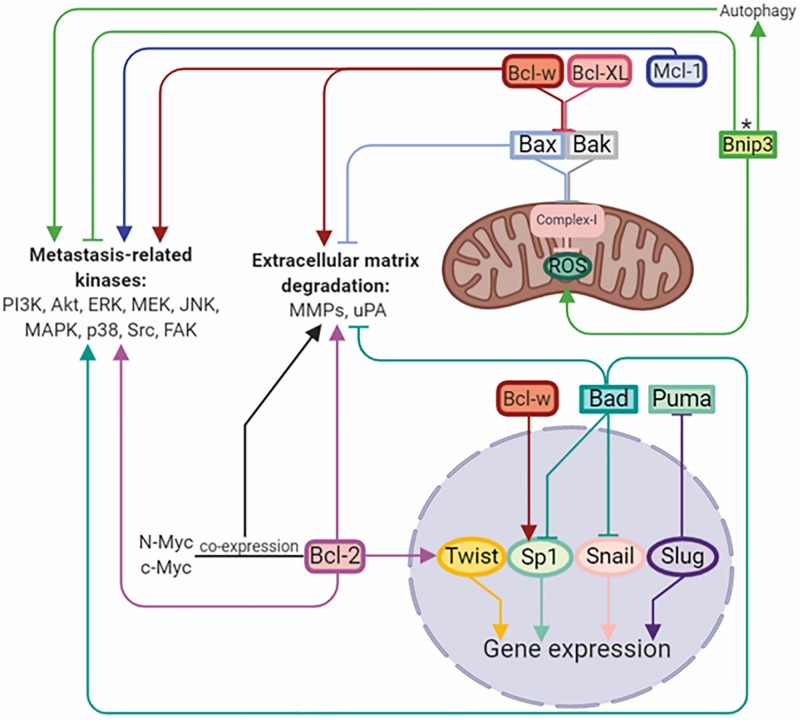
Bcl-2 family members regulate metastasis by activation/inhibition of signaling kinases, matrix-degrading enzymes, and transcriptional factors. Arrows or blunt ends indicate activation or inhibition, respectively. Colored lines corresponds to each protein. ^∗^ - function depends on the tumor type. Bcl-2, B-cell lymphoma 2; Mcl-1, myeloid cell leukemia 1; Bcl-XL, B-cell lymphoma-extra large; Bcl-w, Bcl-2-like protein 2; Bnip3, BCL2/adenovirus E1B 19 kDa protein-interacting protein 3; Bax, Bcl-2-like protein 4; Bak, Bcl-2 homologous antagonist/killer; Bad, Bcl-2-associated death promoter; Puma, p53 upregulated modulator of apoptosis; Twist, class A basic helix-loop-helix protein 38; Sp1, specificity protein 1; Snail, zinc finger protein SNAI1; Slug, zinc finger protein SNAI2. For details, see text. Figure is created using BioRender.

B-cell lymphoma/leukemia gene 2 (Bcl-2) family proteins are considered to be regulators of the apoptotic mitochondrial pathway. This family includes both anti-apoptotic Bcl-2, Bcl-XL, Bcl-w, Mcl-1, Bcl-B, and pro-apoptotic multidomain Bax and Bak proteins. In addition, the pro-apoptotic subfamily includes so-called BH3-only domain proteins, such as Bim, Puma, Noxa, Bad, Bid, and Bnip3. The ratio between these proteins with opposite functions determines the success of apoptosis (Adams and Cory, 2018). Upregulation of anti-apoptotic and downregulation of pro-apoptotic proteins is a hallmark of cancer, and their misbalance is contributed to the chemo-, immune-, and radio-resistance of anticancer therapies (Opferman and Kothari, 2018). However, further evidence has demonstrated that the functions of Bcl-2 family proteins are not limited to cell death control and tumor resistance. It has been established, that Bcl-2 family proteins play crucial roles in the regulation of migration, invasion and metastasis (Um, 2016) (Figure 2).

Indeed, the overexpression of Bcl-2, Bcl-XL, Bcl-w, and Mcl-1 in different cancers, including glioma, neuroblastoma, melanoma, squamous carcinoma, and breast, lung, and colorectal cancer cells, leads to significant increase in their migratory and invasive properties (Sun et al., 2011; Lee et al., 2013; Um, 2016; Young et al., 2016; Trisciuoglio et al., 2017). On the other hand, downregulation of these proteins attenuates invasiveness without affecting apoptosis or tumor growth, indicating that their pro-survival functions are not linked to regulation of cell migration and invasion (Um, 2016). Importantly, several studies have demonstrated that overexpression of the Bcl-2 family proteins is not always sufficient to induce pro-invasive properties of cancer cells, and could require the co-expression of other proteins stimulating invasiveness, such as c-Myc (Lu and Hong, 2009), N-Myc (Noujaim et al., 2002), or Twist1 (Sun et al., 2011). In certain cases, the exposure to hypoxic conditions may also be essential (Trisciuoglio et al., 2005). It has been suggested that the pro-invasive activity of pro-survival Bcl-2 family members appears to vary depending on the cell type and environment (Um, 2016).

The mechanisms involved in Bcl-2 proteins-mediated regulation of invasiveness and metastasis remain incompletely understood. Anti-apoptotic Bcl-2 family proteins have been shown to activate different signaling pathways controlling migration, invasiveness and metastasis ability in cancer. Indeed, Bcl-2 may modulate the EMT program by direct interaction with Twist1 through the helix-loop-helix DNA binding domain of Twist1 and two domains of Bcl-2 in hepatocellular and oral squamous cells (Sun et al., 2011; Duan et al., 2017). In addition, almost all anti-apoptotic Bcl-2 family members regulate the PI3K pathway involved in metastasis progression (Um, 2016). Interplay between Bcl-2 and the p110α subunit of PI3K regulates human colorectal cancer cell migration through actin polymerization and filopodia formation (Wan et al., 2015). Likewise, in lung cancer cells, Bcl-XL increases PI3K and p38 MAPK activities, which subsequently stimulate MMP-2 expression via Akt (Ho et al., 2010). Specifically, Bcl-w was shown to affect migration and invasion pathways through regulation of PI3K, EGF, Src, MMP-2, uPA, and focal adhesion kinase (FAK) (Um, 2016). Furthermore, Bcl-w promotes migration and invasion of glioblastoma cells through β-catenin signaling via its translocation into the nucleus to act as transcription factor for MMP-2 and mesenchymal marker expression (Lee et al., 2013). Additionally, Mcl-1 supports breast cancer cell migration and invasion via Src family kinases (SFKs) and their targets, and also by alteration of the phosphorylation state of the cytoskeletal protein cofilin (Young et al., 2016).

It has been reported that Bcl-2 family members are capable of regulating the functioning of mitochondria, during cellular respiration and of stimulating ROS generation in the form of the superoxide anion radical O_2_^–^ and H_2_O_2_ (Um, 2016). Thus, multidomain pro-apoptotic proteins Bax and Bak have been shown to bind to the ETC Complex-I resulting in decreased ROS production, whereas anti-apoptotic Bcl-w and Bcl-XL interact with Bax and Bak, and abolish their binding to the Complex-I, stimulating ROS production and PI3K-, Src-, and EGFR-dependent cell migration and invasion (Jung et al., 2018; Kim et al., 2018).

Analysis of patients’ clinical samples confirmed the involvement of pro-survival Bcl-2 family members in metastasis. Bcl-2 expression is associated with lymph node metastases of bladder (Kiss et al., 2015) and gastric (Geng et al., 2013) cancer, liver metastases of colorectal cancer (Ishijima et al., 1999) and lymphovascular invasion of patients with breast cancer (Neri et al., 2006). Upregulation of Bcl-XL was observed in lymph node metastases and venous permeation in colorectal cancer (Jin-Song et al., 2011), hematogenous metastases of osteosarcoma patients (Wang et al., 2010) and lymph node metastases in oral tongue squamous cell carcinoma (Zhang et al., 2014). Overexpression of Bcl-w is associated with the infiltrative morphotypes of gastric cancer (Lee et al., 2003) and is overexpressed in patients with lung and breast cancers (Kim et al., 2019). Mcl-1 is upregulated among III–IV a stage of esophageal squamous cell carcinoma patients with lymph node metastases (Xu et al., 2017).

Conversely, pro-apoptotic proteins of this family may suppress cancer cell invasion and metastatic dissemination. Thus, Bax and Bad expression is associated with downregulation of MMP-2, -9, and -10 (Lee et al., 2010; Cekanova et al., 2015). Interestingly, Bax and Bid may downregulate tumor cell invasiveness, indirectly repressing the gene expression of c-Jun, cyclin D1, β-catenin, and Sp1, which are known to stimulate invasive properties and metastasis in breast cancer (Cekanova et al., 2015). In addition, Bad, Bim, and Puma were shown to suppress EMT, inhibiting related transcription factors, including Snai1, Sp1, Snai2, and Slug, and subsequent upregulation of epithelial phenotype markers (Kim et al., 2014; Cekanova et al., 2015; Merino et al., 2015).

Another member of the Bcl-2 family implicated in cancer cell migration, invasion and metastasis is Bnip3 (Maes et al., 2014; Chourasia et al., 2015), which is considered to be a pro-apoptotic Bcl-2 protein. Bnip3 also plays an important role in autophagy and mitophagy regulation (Chourasia et al., 2015). However, its role in cancer progression and metastasis remains highly controversial. Thus, in human triple-negative breast cancer (TNBC) the lack of Bnip3 results in tumor progression and metastasis via storage of dysfunctional mitochondria and subsequent ROS accumulation; the events that, as was discussed earlier, lead to expression of HIF-inducible genes including metastasis-related angiogenesis genes (Chourasia et al., 2015). Conversely, in melanoma cells Bnip3 silencing reduces the formation of lamellipodia and filopodia as well as cell migration through the downregulation of integrin-associated glycoprotein CD47, Rac1 and Cdc42 (Maes et al., 2014). Bnip3 in HCC may suppress metastasis through the JNK/Bnip3/SERCA/CaMKII axis, leading further to the cofilin/F-actin/lamellipodia inhibition (Shi et al., 2018). As mentioned above, Bnip3 is involved in autophagy, the process, which is tightly linked to EMT, migration and metastasis. Specifically, Bnip3-dependent autophagy via hypoxia-induced ROS-modulated p38 MAPK and JNK activation contributes to keratinocytes migration (Zhang et al., 2019). In NSCLC Bnip3 supports metastasis via its modulation by aryl hydrocarbon receptor (AhR), which has an impact on Bnip3 proteasomal degradation and subsequent autophagy disturbance. These events result in decreased EMT progress (Tsai et al., 2017). Thus, Bnip3, depending on cancer cell type and hypoxic conditions, fulfills opposite functions in metastasis.

Analysis of clinical samples confirmed the participation of Bcl-2 pro-apoptotic members in metastasis formation. Increased expression of Bax, Bak and Puma is associated with a lack of vascular invasion in patients with oral squamous cell carcinoma (Coutinho-Camillo et al., 2010). Loss of Bax protein was demonstrated in retinoblastoma specimens with massive choroidal invasion (Singh et al., 2015). Downregulation of Bnip3 is characteristic of lymph node metastases in breast cancer (Koop et al., 2009). Conversely, in patients with renal cell carcinomas Bnip3 expression is correlated with lymph node metastasis (Macher-Goeppinger et al., 2017). Methylation of Bim and Bnip3 genes is associated with metastasis and the gene methylation rate is increased among colorectal and pancreatic cancer patients compared to healthy individuals (Shimizu et al., 2010; Mhaidat et al., 2017; Zhu et al., 2017). Interestingly, overexpression of pro-apoptotic Bcl-2 family members, except Bnip3, frequently correlates with decreased metastasis and favorable outcomes in patients with various cancer types (Chi et al., 2016).

Thus, anti-apoptotic members of the Bcl-2 family, including Bcl-2, Bcl-XL, Bcl-w, and Mcl-1, support the invasion and metastasis in various types of cancer. This positive influence is achieved through EMT, subsequent cytoskeleton rearrangement, overexpression of MMPs and uPA, and regulation of PI3K, p38 MAPK, Akt, and ERK. In contrast to anti-apoptotic proteins, almost all pro-apoptotic members, such as Bax, Bak, Bad, Bid, and Puma, were characterized by suppression of the metastatic potential of cancer cells. Despite Bnip3 being considered a pro-apoptotic member, its influence on metastasis remains highly controversial, likely due to its atypical BH3-domain contributing to autophagy-dependent processes (Mazure and Pouysségur, 2009). Bcl-2 family members play important role in the regulation of ROS production and activity of mitochondrial complexes leading to the activation of molecular pathways controlling invasion and metastasis. Moreover, the Bcl-2 family proteins affect cell migration of both malignant and normal tissues. Analysis of patient specimens with tumors confirms the participation of Bcl-2 family members in invasion and metastasis, which gives a reason to consider these proteins for target therapy.

## ER-Mitochondria Network and Metastasis

The endoplasmic reticulum (ER) is a crucial cellular Ca^2+^ reservoir, that coordinates Ca^2+^ signaling, protein synthesis and folding and traffic of properly folded proteins to the Golgi apparatus. Accumulation of misfolded proteins in the ER lumen triggers unfolded protein response (UPR), which is an adaptive signaling pathway to restore protein homeostasis (proteostasis). If accumulation of misfolded proteins remains unresolved activation of UPR signaling may lead to the initiation of apoptotic cascades. Crosstalk between apoptosis and UPR is maintained by mitochondria functioning partly due to contact sites with ER. These so-called mitochondria-associated ER membranes (MAMs) are key for Ca^2+^ transport between the ER and mitochondria to maintain cellular homeostasis and regulate ER stress. Moreover, MAMs form functional networks essential in determining pro-survival/pro-death and inflammation signaling (Malhotra and Kaufman, 2011).

As mentioned above, ER is a multifunctional organelle, the main function of which is to control protein-folding quality. Numerous factors may affect proper protein folding in the ER, including oxidative stress, hypoxia, glucose deficiency, viral infections and other physical/chemical stresses. As a result, it leads to ER stress and subsequent UPR. In mammals the UPR is carried out by three distinct ER-related transmembrane proteins, including protein kinase RNA-like ER kinase (PERK), endoribonuclease inositol-requiring enzyme 1 alpha (IRE1α/IRE1), and activating transcription factor 6 (ATF6) (Malhotra and Kaufman, 2011). In unstressed cell, ER-related transmembrane proteins including PERK, IRE1α/IRE1, and ATF6 are bound to immunoglobulin heavy chain protein/glucose-regulated protein 78 (BiP/GRP78). Under ER stress BiP dissociates from these proteins to trigger signaling pathways that result in the reduction of global protein synthesis, degradation of unfolded proteins and increase of protein-folding capacity of the ER (Lee, 2005).

Endoplasmic reticulum plays an important role in mitochondrial calcium signaling via the contact sites between mitochondria and ER (MERCs). The portion of membranes involved in these interactions defines the MAMs, which, as mentioned above, provide Ca^2+^ traffic between these organelles (Martinvalet, 2018). The transport of extracellular Ca^2+^ into the cytosol occurs through the voltage-gated, ligand-gated, and store-operated Ca^2+^-channels (SOCCs) including Orai and/or transient receptor potential channels (TRPC). Orai1 and TRPC are activated through their binding to the stromal interaction molecule 1 (STIM1), which is the ER Ca^2+^-sensor. Ca^2+^ transport from the cytosol and its accumulation in the ER depends on the action of ATP-driven sarco/ER Ca^2+^-ATPase (SERCA) (Bower et al., 2017). Ca^2+^ is transported from the ER via 1,4,5-trisphosphate (IP3) and ryanodine receptors (IP3Rs, RyRs), after which Ca^2+^ invades the mitochondria through the voltage-dependent anion channels (VDACs) on the OMM (Báthori et al., 2006). Ca^2+^ then is transferred by the mitochondrial calcium uniporter (MCU) on the IMM (Martinvalet, 2018). Expression levels of these calcium-signaling proteins are frequently altered in numerous types of tumor cells (Singh et al., 2017) and most of all govern metastasis-related processes.

As mentioned above, ER is the main cellular Ca^2+^-store. Decreased Ca^2+^ level in the ER results in STIM1 oligomerization and its transfer from ER to the plasma membrane where it promotes Orai1-dependent store-operated Ca^2+^ entry (SOCE) (Yang, 2018). A growing body of evidence indicates the existence of an interplay between mitochondria and SOCE. Indeed, SOCE activation is accompanied by TRPC-modulated increase in cytosolic Na^+^ level that in turn promotes the activation of mitochondrial Na^+^/Ca^2+^ exchanger (NCLX) leading to mitochondrial Na^+^ influx and Ca^2+^ efflux. Therefore, NCLX tightly regulates mitochondrial Ca^2+^ level and prevents excessive Ca^2+^ accumulation in mitochondria that can lead to the increase of the mtROS level and subsequent SOCE suppression via oxidation of redox-sensitive Cys195 of Orai1 (Ben-Kasus Nissim et al., 2017). Besides NCLX, the activity of SOCE-related proteins is regulated by Bcl-2. The mutations in BH1 domain of Bcl-2 protein leads to STIM1, Orai1-3, TRPC1 overexpression and SOCE enhancement (Chiu et al., 2018). It has been established, that hyperactive SOCE induced by STIM1 and Orai1 overexpression correlates with increased metastasis in different types of cancer. Intensified SOCE supports tumor cell invasion and migration by cytoskeleton rearrangement, ECM degradation and tumor microenvironment remodeling (Yang, 2018).

Sarco/ER Ca^2+^-ATPase is a well-known regulator of Ca^2+^ stores in the ER and maintains the level of Ca^2+^ uptake and leak properties. Furthermore, SERCA inactivation associated with Yap deficiency has been shown to inhibit HCC metastasis through the cofilin/F-actin/lamellipodium pathway (Shi et al., 2018). Additionally, the downregulation of SERCA leads to a significant decrease in Ca^2+^ level in migrating cells that in turn inhibits cell migration and tracheogenesis (Bower et al., 2017). Other proteins localized to MAMs are VDAC and IP3R, the action of which is dependent on Bcl-2 family proteins and, in addition to their role in apoptosis, partly regulate Ca^2+^ signaling via their complex with MAM proteins (Monaco et al., 2015; Bittremieux et al., 2019). Anti-apoptotic family members Bcl-2, Bcl-XL, and Mcl-1 bind to VDACs and suppress mitochondrial Ca^2+^ transport that in turn supports cell migration and invasion (Huang et al., 2013, 2014; Fouqué et al., 2016). Both Bcl-2 and Bcl-XL interact with VDAC1 through BH4 domain; however, Bcl-XL BH4 is more effective than Bcl-2-BH4 in targeting VDAC1 activity (Monaco et al., 2015). Dissociation between these anti-apoptotic Bcl-2 family members and VDACs results in decreased migration of TNBC (Fouqué et al., 2016) and NSCLC cells (Huang et al., 2014). Bcl-2 family proteins could also interact with IP3R suppressing Ca^2+^-release. Like VDACs, Bcl-2 binds IP3R through its BH4 domain inhibiting its activity. Besides Bcl-2, other anti-apoptotic family members, including Bcl-XL and Mcl-1, are able to influence IP3R activity and Ca^2+^ signaling, but their role in mitochondria-associated ER membrane-related calcium signaling still remains controversial (Eckenrode et al., 2010; Monaco et al., 2012). Also like VDACs, IP3R may regulate cell migration separately from its complex with Bcl-2 family members. It has been revealed, that inhibition of ryanodine receptor subtype IP3R3 and subsequent decrease in Ca^2+^ release results in suppression of the invasion and migration of glioblastoma cell lines and metastasis in glioblastoma mouse model (Kang et al., 2010). Overexpression of IP3R3, but not of IP3R1 and IP3R2, leads to stimulation of the migration properties of breast cancer cells sustaining Ca^2+^ signaling (Mound et al., 2017). Thus, IP3R could regulate cancer cell migration and metastasis through modifying calcium ER level.

**FIGURE 3:**
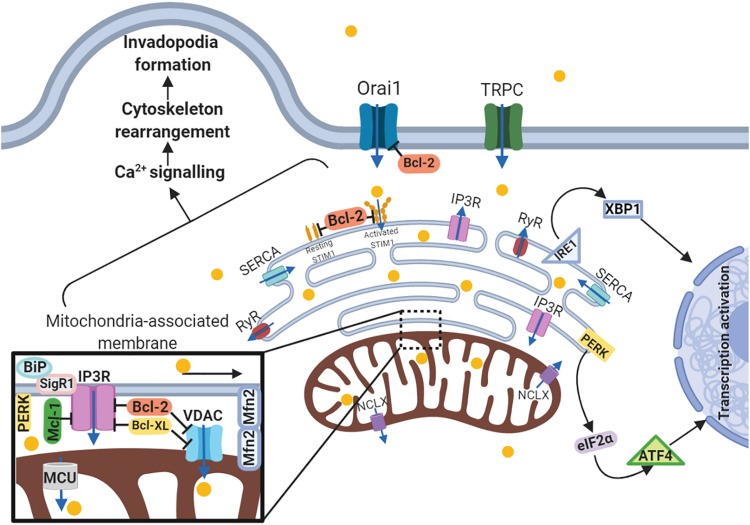
Schematic representation of the link between ER-mitochondria network and motility of cancer cells. Arrows or blunt ends indicate activation or inhibition, respectively. Blue arrows indicate direction of Ca^2+^ current. Yellow circles – Ca^2+^. MAM, mitochondria-associated ER membrane; Orai1, calcium release-activated calcium channel protein 1; TRPC, transient receptor potential cation channel; SERCA, sarco/endoplasmic reticulum Ca^2+^-ATPase; STIM1, stromal interaction molecule 1; IP3R, inositol trisphosphate receptor; RyR, ryanodine receptor; IRE1, serine/threonine-protein kinase/endoribonuclease inositol-requiring enzyme 1; XBP1, X-box binding protein 1; PERK, protein kinase RNA-like endoplasmic reticulum kinase; eIF2α, eukaryotic translation initiation factor 2; ATF4, activating transcription factor 4; NCLX, mitochondrial Na^+^/Ca^2+^ exchanger; BiP, binding immunoglobulin protein; SigR1, sigma receptor 1; Mfn2, mitofusin2; VDAC, voltage-dependent anion-selective channel; MCU, mitochondrial calcium uniporter. For details, see text. Figure is created using BioRender.

Another MAM-related protein is Sig1R (stress-activated chaperone sigma-1 receptor). When ER stress is not activated, Sig1R cooperates with MAMs chaperone BiP/GRP78, whereas under activation of IP3Rs Sig1R dissociates from chaperone BiP and binds to IP3R3, leading to its stabilization at the MAM and increasing Ca^2+^ flux to the mitochondria (De Pinto and Palmieri, 1992; Naon and Scorrano, 2014). The expression level of MAM-associated Sig1R is increased in metastatic breast and colorectal cancer cells as compared to normal tissues (Gueguinou et al., 2017). Consistently with the above-mentioned MAM-related proteins, MCU also affects migration, invasion and metastasis. Silencing of this uniporter results in decreased mitochondrial Ca^2+^ level and ROS production, as well as migratory and invasiveness capacities. These findings are in good agreement with *in vivo* experiment. MCU gene deletion reduces tumor metastasis in TNBC MDA-MB-231 xenografts via HIF-1-dependent gene expression (Tosatto et al., 2016). Yu et al. (2017) demonstrated similar results in breast cancer MCF-7 cells by MCU overexpression, which leads to enhanced migratory and invasiveness potential *in vitro* and lung metastasis mouse model *in vivo*. Furthermore, overexpressed MCU was found in specimens from breast cancer patients with metastases. Thus, MCU expression correlates with migration and invasion of cancer cells, as well as with tumor metastasis, which has been proved by both *in vitro* and *in vivo* studies. Besides MAM-anchored proteins, UPR regulators, including PERK, IRE1 and BiP/GRP78, all influence the migration and metastasis. PERK as a key UPR sensor, also participates in MAM signaling (Verfaillie et al., 2012). A growing amount of evidence proves that UPR signaling and EMT reprogramming mutually activate each other. In gastric cancer cells knockdown of UPR-related proteins such as PERK, ATF4, and ATF6, decrease TGF-β expression and abrogates EMT under severe hypoxia (Shen et al., 2015). The inverse pattern in this UPR-EMT axis has been demonstrated in both *in vivo* and *in vitro* models of breast cancer: cells undergoing EMT have a branched ER structure and activated PERK–eIF2α link of the UPR, which helps cells to metastasize. Analysis of specimens from patients with breast, gastric, colon and lung metastatic tumors revealed correlations between expression of EMT and PERK–eIF2α genes (Feng et al., 2014). Additionally, ATF4, ATF6, another ER-transmembrane protein IRE1 and its-related X-box binding protein-1 (XBP1) play a role in metastatic progression. IRE1α regulates actin cytoskeleton rearrangement and influences the cell migration via filamin A in MEFs, fly, and zebrafish models (Urra et al., 2018). IRE1-XBP1 pathway is regulated by lysyl oxidase-like 2 (LOXL2) overexpression, and activates EMT via Snai1/2, ZEB2 and TCF3 transcription factors in breast carcinoma cells (Cuevas et al., 2017). Notably, XBP1 expression is significantly upregulated in tumor and lymph node metastases compared to normal tissues from patients with oral squamous cell carcinoma, and downregulated XBP1 expression results in decreased cell invasion capacity (Sun et al., 2018c). Thus, proteins of three distinct UPR branches, including PERK, ATF4, ATF6, IRE1, XBP1, and BiP/GRP78, contribute to cancer cell invasive properties and metastatic dissemination regulating MAM signaling (Figure 3).

## Metabolism and Metastasis

Altered metabolic activity is one of the hallmarks of cancer. Cancer cells change their metabolism in order to satisfy increasing of bioenergetic and biosynthetic demand and maintain tumor growth (DeBerardinis and Chandel, 2016). Unlike normal cells, which generate much of their ATP via mitochondrial-dependent OXPHOS, cancer cells often demonstrate upregulation of glycolysis even under conditions when oxygen concentration is not limited (Lehuédé et al., 2016; Teoh and Lunt, 2018). This phenomenon was observed in the 1920s by Otto Warburg, who demonstrated that tumor tissues metabolize approximately ten-fold more glucose to lactate in a given time than do normal tissues, which led him to conclude that cancer cells rely on glycolysis more than do healthy cells. Enhanced aerobic glycolysis has been detected in many types of cancer and is correlated with worse clinical outcome (Yu et al., 2019). However, further studies have demonstrated that cancer cells may also engage mitochondrial respiration in addition to glycolysis (Jia et al., 2018). Indeed, breast cancer cells produce most of their ATP through mitochondrial oxidation (Park et al., 2016). Similarly, glioma cell lines are strongly dependent on mitochondrial OXPHOS for ATP production (Griguer et al., 2005). Moreover, cancer cells may display distinct metabolic characteristics depending on the tissue of origin (Elia et al., 2015). Thus, lung, liver and colorectal cancers, and leukemias depend on glycolysis, whereas, melanomas, lymphomas, and glioblastomas are characterized as oxidative tumors (Elia et al., 2015; Obre and Rossignol, 2015; Lehuédé et al., 2016). Tumor cells can also switch from one type of metabolism to another under glucose-limiting conditions as observed in cervical cancer, breast carcinoma, hepatoma and pancreatic cancer cells (Rossignol et al., 2004; Beckner et al., 2005; Jose et al., 2011; Smolková et al., 2010). A growing number of studies provide the evidence that cancer cell migration is associated with significant metabolic alterations supporting metastatic dissemination (Morandi et al., 2017; Teoh and Lunt, 2018). Thus, it was reported that increased motility of cancer cells requires the shift toward utilization of glycolytic pathways (Shiraishi et al., 2015). Glycolytic genes activation has been detected in different tumors and is often associated with malignant and aggressive phenotypes (Jose et al., 2011). For example, expression of hexokinase 2 (HK2), the embryonic isoform of hexokinase, the enzyme which defines the start of glycolysis, is associated with increased risk of recurrence, and adverse clinical outcome for breast cancer, pancreatic cancer, and neuroblastoma patients (Teoh and Lunt, 2018). Further studies have demonstrated that glycolytic enzymes also contribute to the metastatic progression of cancer cells (Teoh and Lunt, 2018). For example, pyruvate kinase isozyme M2 (PKM2), which mediates the final rate-limiting step of glycolysis, promotes aggressive phenotype and metastasis in different types of tumors (Zhou et al., 2012; Yu et al., 2015; Teoh and Lunt, 2018). This enzyme also acts as a transcriptional coactivator of HIF-1α in cancer cells, thus promoting glycolysis and inducing EMT (Xu et al., 2012; Morandi et al., 2017). Furthermore, EMT stimulation induced by TGF-β leads to the nuclear translocation of PKM2 in colon cancer cells, where it interacts with TGIF2 and other transcription factors, promoting EMT and supporting the malignant properties of tumor cells (Hamabe et al., 2014). Phosphohexose isomerase (PHI) is another glycolytic enzyme that involved in stimulation of invasion and metastatic dissemination through extracellular autocrine motility factor (AMF) (Watanabe et al., 1996). The overexpression of PHI leads to the increased invasion and metastasis of colon cancer cells (Tsutsumi, 2009). Additionally, PHI/AMF overexpression has been reported to promote the EMT activation through the NF-κB pathway and increased expression of EMT markers such as Snai1 and ZEB1/2 (Ahmad et al., 2011). In keeping with these observations, high PHI levels in the serum correlate positively with metastases of colorectal and esophageal squamous cells, and lung tumors (Nakamori et al., 1994; Takanami et al., 1998). Conversely, downregulation of glycolytic enzymes including PKM2 and PHI, inhibit the proliferation and migration of cancer cells (Zhou et al., 2012; Teoh and Lunt, 2018). Inhibition of glycolysis attenuates cell motility even while mitochondrial ATP synthesis remains intact, and inhibition of mitochondrial respiration reduces cell motility only minimally compared to inhibition of glycolysis (Shiraishi et al., 2015).

Glycolysis regulates different stages of metastatic dissemination, contributing to the different stages of the metastatic cascade. Thus, prostate cancer cells undergoing EMT and acquiring mesenchymal features exhibit higher glycolytic activity than their epithelial counterparts. High glycolysis rate is associated with increased cytoskeletal rearrangement and cell migration. In turn, inhibition of glycolysis suppresses the migration properties of prostate cancer cells (Shiraishi et al., 2015). In addition, an interrelation between EMT induced by TGF-β, activation of the glycolytic pathway, and repression of mitochondrial function was demonstrated (Morandi et al., 2017). In breast cancer, loss of fructose-1,6-bisphosphatase together with the loss of E-cadherin promotes cancer stem cell (CSC)-like features and cancer cell dissemination by enhancing β-catenin signaling and the EMT program. These events are concomitant with the induction of glycolysis, increase in glucose uptake, and inhibition of oxygen consumption (Dong et al., 2013).

Although it is known that metastasis requires activation of the glycolytic program, recent studies demonstrate an equal importance of OXPHOS for metastatic dissemination (Porporato et al., 2018). For example, the strong correlation between expression of PGC-1*a* (peroxisome proliferator-activated receptor gamma coactivator-1*a*), a key regulator of mitochondrial biogenesis and OXPHOS, and invasive properties was observed in breast cancer cells (LeBleu et al., 2014). PGC-1*a* supports migration of cancer cells, stimulating mitochondrial biogenesis and respiration, whereas downregulation of PGC-1*a* decreases the frequency of metastasis. Elevated OXPHOS activity is also linked to the high metastatic potential in mouse melanoma and human cervical cancer cells (Porporato et al., 2014). In turn, prostate cancer cells exhibit a mixed phenotype, where both glycolysis and OXPHOS are required for energy metabolism at different stages of disease progression (Costello and Franklin, 2005). This hybrid metabolic state, also called metabolic plasticity, can sustain tumor cell survival under different micro-environmental conditions, while at the same time supporting tumor metastasis and therapy-resistance. Thus, a hybrid metabolic phenotype, characterized by high HIF-1/AMPK activities and high glycolysis/OXPHOS (glucose oxidation and FAO) activities, allows cancer cells to acquire metabolic plasticity and utilize different types of nutrients (Jia et al., 2019). Furthermore, it permits the cells to produce energy efficiently through multiple metabolic pathways and meanwhile synthesize biomass for rapid proliferation using by-products from glycolysis. A hybrid metabolic phenotype maintains the cellular ROS at a moderate level so that cancer cells can benefit from ROS signaling and avoid DNA damage due to excessive ROS (Vyas et al., 2016).

Finally, different metabolic profiles may dictate metastatic fitness to distinct organ sites (Lehuédé et al., 2016). It has been shown that metastatic breast cancer cells may display different metabolic pathways depending on the site of metastasis. Hence, breast cancer cells obtained from bone and lung metastases rely on OXPHOS, whereas liver-metastatic breast cancer cells engage a glycolytic type of metabolism (Dupuy et al., 2015).

In consequence, metabolic pathways of migrating cancer cells appear to be inter-connected and characterized by plasticity depending on different factors, i.e., tumor type, microenvironment, site of metastasis formation, etc. A better understanding of this metabolic plasticity will permit the design of specific therapy approaches in order to target metastatic cancer cells more efficiently.

## Therapeutic Targeting Metastasis

Metastasis is associated with poor outcome of cancer patients (Porporato et al., 2014). Existing therapeutic approaches are often ineffective or provide limited clinical benefit. Hence, mitochondria play an important role in metastatic dissemination, the targeting mitochondria might represent an attractive approach for the development of new strategies for treatment of metastatic cancers.

Metastatic tumors have been shown to reprogram their metabolism in order to successfully metastasize (Lehuédé et al., 2016). Accordingly, significant efforts have been made to target cancer cell metabolism in different tumors for the prevention of metastasis progression. For example, the anti-diabetic drug metformin has been shown to possess anticancer properties in different types of cancer (Rattan et al., 2011; Schexnayder et al., 2018; Thakur et al., 2018). Metformin is a Complex I inhibitor, providing cancer metabolism suppression through downregulation of mitochondrial glycerophosphate dehydrogenase (mGPDH) and OXPHOS inhibition, leading to decreased metastasis levels in a thyroid cancer mouse model (Thakur et al., 2018). Metformin also attenuates the growth of lung metastatic nodules in an ovarian cancer mouse model by inhibiting the mTOR signaling pathway (Rattan et al., 2011). At low concentrations metformin inhibits breast cancer invasion and metastasis by suppressing ROS production, suggesting the use of metformin as a chemopreventive agent to block cancer cell invasiveness (Schexnayder et al., 2018). Although, the precise mechanisms of action of metformin are still debated, a number of clinical studies have confirmed its antitumor properties (Pollak, 2012). At present metformin is used during the treatment of different cancer types in order compound to inhibit hypoglycemia non-target effect of various chemotherapy drugs (da Veiga Moreira et al., 2019). Further clinical studies are required to clarify its anti-metastatic properties.

Glycolysis inhibition has been shown to suppress metastasis in several types of cancer (Caino and Altieri, 2016). For example, HKII inhibitor lonidamine (TH-070), a derivative of indazole-3-carboxylic acid, provided significant effectiveness in preclinical studies when the drug was administered in combination with paclitaxel and cisplatin (Sborov et al., 2015; Caino and Altieri, 2016). However, despite promising early stage results, further phase II and phase III trials targeting lung cancer with lonidamine have shown its limited efficacy and hepatic toxicity (Cervantes-Madrid et al., 2015). Importantly, a more recent study demonstrated that the use of modified lonidamine is significantly more efficacious in inhibiting mitochondrial bioenergetics in lung cancer cells, leading to suppression of lung cancer progression and metastasis. Mitochondrial-lonidamine activates the generation of ROS in lung cancer cells, which leads to the inactivation of the Akt/mTOR/p70S6K signaling pathways and autophagic cell death (Cheng et al., 2019). Glycolysis can be targeted by 2-deoxyglucose (2-DG), a non-metabolizable glucose analog, which is also pursued in the clinic. However, dose-escalation phase I trials in patients with castrate-resistant prostate cancer and other advanced solid tumors resulted in asymptomatic QTc prolongation that limited further drug evaluation (Sborov et al., 2015). Since tumors may shift from glycolysis to OXPHOS, or even engage hybrid metabolisms, several studies have proposed the dual inhibition of cancer metabolism using metformin and 2-DG (Cheong et al., 2011; Jia et al., 2019). Indeed, combined treatment with metformin and 2-DG led to the significant suppression of tumor growth and metastasis in preclinical models (Cheong et al., 2011).

Mitochondrial ROS have been reported to function as signaling molecules implicated in the regulation of tumor growth and metastasis (Porporato et al., 2014). The different mechanisms by which ROS contribute to tumor growth and metastatic dissemination were discussed above. Thus, targeting mtROS seems to be an attractive approach for cancer therapy. However, contrary to the expected results, the use of antioxidants for anticancer treatment led to increased risk of cancer (Klein et al., 2011; Sullivan and Chandel, 2014). Furthermore, the treatment with NAC was shown to enhance the metastatic dissemination of human melanoma cells, providing evidence that oxidative stress may, in certain circumstances, stimulate metastasis (Piskounova et al., 2015). The cause of the failure of treatment with antioxidants could be their lack of specificity. They also may regulate many different processes involved in tumor growth and metastasis (Sullivan and Chandel, 2014). On the other hand, it has been shown that inhibition of ROS with antioxidants that target precisely mitochondrial oxidative stress may stop metastatic spread. Scavenging with MitoTempo, specific mitochondrial antioxidant, significantly reduced cancer cell invasion and prevented metastasis (Porporato et al., 2014). Thus, ROS targeting appears to be more complex than believed before and requires further detailed investigation.

Another therapeutic agent that has shown promising results in preclinical studies is an inhibitor of mitochondrial heat shock protein 90 (Hsp90) Gamitrinib (Kang et al., 2011). This compound induces mitochondrial dysfunction, providing depolarization of inner membrane potential that in turn regulates the release of cytochrome *c*. In mouse model of prostate cancer, Gamitrinib administration inhibited tumor growth and metastasis affecting mitochondria (Kang et al., 2011). Furthermore, targeting Hsp90 with Gamitrinib suppresses cancer cell migration and metastasis preventing metabolic reprogramming and increasing AMPK phosphorylation (Caino et al., 2013), Gamitrinib is also effective in combination therapies with inhibitors of both TRAIL and PI3K (Siegelin et al., 2011; Ghosh et al., 2015).

Since the expression of Bcl-2 family proteins has been detected in metastases of different tumors, another possible approach for cancer therapy may be focused on targeting Bcl-2 family members. BH3-mimetics are promising therapeutic drugs that mimic endogenous Bcl-2 family member antagonists, thereby target some of them and abrogating their anti-apoptotic functions. Initially, BH3-mimetics displayed encouraged results in hematological malignancies including lymphoma lymphocytic leukemia, acute myeloid leukemia, small lymphocytic lymphoma and mantle-cell lymphoma (Cang et al., 2015; Mullard, 2016; DiNardo et al., 2018; Tam et al., 2018). Thus, first-generation BH3-mimetics such as ABT-737 and its orally available derivative navitoclax (ABT-263), which are inhibitors of Bcl-2 and Bcl-W, have shown clinical efficacy (Billard, 2013). However, in several cases the treatment with these agents was limited by severe thrombocytopenia (Mullard, 2016). Clinical studies of ABT-199 in chronic lymphocytic leukemia and non-Hodgkin’s lymphoma have shown impressive antitumor efficacy, with higher response rates than navitoclax and without thrombocytopenia (Besbes et al., 2015). Several clinical trials have also demonstrated the efficacy of BH3-mimetics in solid tumors (Boisvert-Adamo et al., 2009; McKee et al., 2013; Mukherjee et al., 2018). In particular Mcl-1 has emerged as a promising target for the treatment of melanoma (Boisvert-Adamo et al., 2009; McKee et al., 2013). Additionally, a novel gossypol derivative and BH3-mimetic ch282-5 (2-aminoethanesulfonic acid sodium-gossypolone) induced colon cancer cell death *in vitro* and *in vivo*. Ch282-5 treatment activated mitochondria-dependent apoptotic pathway accompanied by mitophagy disruption and mTOR pathway activation. Furthermore, Ch282-5 provided suppression of colon cancer cell migration, invasion and liver metastasis (Wang et al., 2016). Notably, Bcl-2 family members were shown to interact with Drp1 and treatment with BH3-mimetic A-1210477 led to Drp1-dependent mitochondria fragmentation, whereas Drp1 silencing significantly reduced apoptosis induced by BH3-mimetic in lung, cervical, and breast cancer cell lines (Milani et al., 2018). Conversely, inhibition of Drp1 in combination with BH3-mimetic treatment significantly enhanced apoptotic response in melanoma cells (Mukherjee et al., 2018). Additionally, inhibition of Drp1 by Mdivi-1 increased the cytotoxic effect of combination treatment with A-1210477 and ABT-263 in different melanoma cell lines (Mukherjee et al., 2018).

Another interesting approach to target metastatic cancers is the regulation of mitochondrial K^+^/H^+^ exchange. Salinomycin is an antibiotic from the polyether ionophores group widely used in agriculture (Managò et al., 2015). Recently, it has been revealed that it possesses anticancer properties in different types of cancer (Klose et al., 2019; Tang et al., 2011). Salinomycin may target chemoresistant tumor cells, inhibiting Wnt/β-catenin and Sonic Hedgehog signaling pathways (Managò et al., 2015). Furthermore, it suppresses the migration of colorectal, breast, lung and colon cancer cell lines as well the invasion of nasopharyngeal carcinoma and bladder cancer cells *in vitro* (Kopp et al., 2014; Wu et al., 2014; Qu et al., 2015; Klose et al., 2016). Consistently, *in vivo* studies proved that salinomycin may reduce metastasis formation in mammary tumor mouse model, bladder tumor rat model and intravenous mouse tumor model (Gupta et al., 2009; Kopp et al., 2014; Qu et al., 2015). Importantly, salinomycin is able to suppress the late stages of autophagy contributing to the ROS generation and mitochondria dysfunction (Klose et al., 2019). This might explain the mechanism by which salinomycin targets mitochondrial K^+^/H^+^ exchange and prevents migration, invasion and metastasis.

Summarizing, mitochondria contribute to tumor progression and metastasis through different mechanisms including redox signaling, mitochondrial biogenesis, regulating Bcl-2 family members, metabolic reprogramming and mitochondrial K^+^/H^+^ exchange. The better understanding of these mechanisms and the possible interplay between them may provide new therapeutic approaches to target metastatic diseases.

## Conclusion

Mitochondria are very important and complex organelles that affect tumorigenesis and metastatic dissemination through different mechanisms including regulation of metabolism, redox status, signaling and cell death pathways. Recent evidence has demonstrated the existence of complex interplay between mitochondria-related functions and mitochondrial dynamics. Thus, dysregulated mitochondrial turnover contributes to tumorigenesis and metastases. However, the mechanisms connecting mitochondrial dynamics to the development of metastasis remain poorly understood. In addition, the flexibility of mitochondria that allow cancer cells to adapt to the changing microenvironment and stresses should be considered in order to combat cancer successfully. Consequently, a better understanding of the processes regulated by mitochondria and their complex interplay with mitochondrial biogenesis may offer new promising therapeutic strategies for cancer treatment.

## Author Contributions

All authors discussed the outline of the manuscript and involved in the preparation of the final version of the manuscript. TD and AG prepared the first draft, figure, and table.

## Conflict of Interest

The authors declare that the research was conducted in the absence of any commercial or financial relationships that could be construed as a potential conflict of interest.
